# In vivo development of immune tissue in human intestinal organoids transplanted into humanized mice

**DOI:** 10.1038/s41587-022-01558-x

**Published:** 2023-01-26

**Authors:** Carine Bouffi, Kathryn A. Wikenheiser-Brokamp, Praneet Chaturvedi, Nambirajan Sundaram, Gillian R. Goddard, Mark Wunderlich, Nicole E. Brown, Janet F. Staab, Rachel Latanich, Nicholas C. Zachos, Emily M. Holloway, Maxime M. Mahe, Holly M. Poling, Simon Vales, Garrett W. Fisher, Jason R. Spence, James C. Mulloy, Aaron M. Zorn, James M. Wells, Michael A. Helmrath

**Affiliations:** 1 Division of Pediatric General and Thoracic Surgery, Cincinnati Children’s Hospital Medical Center, Cincinnati, OH USA; 2 Division of Pathology and Laboratory Medicine, Cincinnati Children’s Hospital Medical Center, Cincinnati, OH USA; 3 Department of Pathology and Laboratory Medicine, University of Cincinnati, Cincinnati, OH USA; 4 Division of Developmental Biology, Cincinnati Children’s Hospital Medical Center, Cincinnati, OH USA; 5 Center for Stem Cell and Organoid Medicine (CuSTOM), Cincinnati Children’s Hospital Medical Center, Cincinnati, OH USA; 6 Division of Experimental Hematology and Cancer Biology, Cincinnati Children’s Hospital Medical Center, Cincinnati, OH USA; 7 Division of Gastroenterology and Hepatology, Department of Medicine, Johns Hopkins University School of Medicine, Baltimore, MD USA; 8 Université de Nantes, Inserm, TENS, The Enteric Nervous System in Gut and Brain Diseases, IMAD, Nantes, France; 9 Department of Pediatrics, University of Cincinnati, Cincinnati, OH USA; 10 Division of Gastroenterology, Department of Internal Medicine, Department of Cell and Developmental Biology, University of Michigan Medical School, Ann Arbor, MI USA; 11 Department of Biomedical Engineering, University of Michigan College of Engineering, Ann Arbor, MI USA

**Keywords:** Tissue engineering, Mucosal immunology, Differentiation

## Abstract

Human intestinal organoids (HIOs) derived from pluripotent stem cells provide a valuable model for investigating human intestinal organogenesis and physiology, but they lack the immune components required to fully recapitulate the complexity of human intestinal biology and diseases. To address this issue and to begin to decipher human intestinal–immune crosstalk during development, we generated HIOs containing immune cells by transplanting HIOs under the kidney capsule of mice with a humanized immune system. We found that human immune cells temporally migrate to the mucosa and form cellular aggregates that resemble human intestinal lymphoid follicles. Moreover, after microbial exposure, epithelial microfold cells are increased in number, leading to immune cell activation determined by the secretion of IgA antibodies in the HIO lumen. This in vivo HIO system with human immune cells provides a framework for future studies on infection- or allergen-driven intestinal diseases.

## Abstract (web summary)

Human intestinal organoids are endowed with immune cells by transplantation into humanized mice.

## Main

The intestine constitutes the largest compartment of the immune system, organized in the lamina propria, epithelium and lymphoid follicles defined by the gut-associated lymphoid tissue (GALT). Immune cell types and GALT are regionally distributed across the mucosal layers and along the gut tube^1^. Immune–epithelial crosstalk is essential to the maintenance of intestinal homeostasis, defense against pathogens and immunologic tolerance to dietary components and commensal bacteria. For instance, specialized epithelial microfold (M) cells, located in the follicle-associated epithelium, play a central role in gut immune sensing by transporting luminal antigens in the lamina propria to activate immune cells^2^. Immune–epithelial crosstalk mediated by cytokines is involved in gastrointestinal tissue development, homeostasis and disease states^3–5^. Although experimental mouse models have been widely used to dissect the biological mechanisms of intestinal immune tissue development and disease, there are still substantial differences that prevent direct extrapolation to human biology^6^. Similarly, coculture of HIOs and immune cells has not fully recapitulated the complexity of human immune intestinal tissue^7–9^. Deeper understanding of the perturbations of the gastrointestinal immune system that commonly result in chronic human diseases requires new human models that represent patient-specific gastrointestinal immune tissue.

During the past decade, pluripotent stem cell (PSC)-derived HIOs have advanced our knowledge of human intestinal development, physiology and diseases^10,11^. A key feature of the PSC-derived HIO model is the ability to generate complex human intestinal tissue. We have previously developed and validated an in vivo model that uses transplantation of in vitro PSC-derived HIOs into immunocompromised mice. We demonstrated the induction of intestinal maturation in transplanted HIOs, characterized by the presence of stem cells and functionally differentiated epithelial cells, as well as a crypt/villus architecture with mesenchymal submucosal layers including lamina propria^12–14^. However, this system lacked the developing immune cells that would normally be found in the developing fetal intestine. To overcome this limitation, here we report a next-generation HIO system containing human immune cells, obtained by growing HIOs in mice with a humanized immune system.

Humanized immune system mouse models are commonly used to investigate human hematopoiesis or inflammatory diseases and can be generated by the engraftment of human peripheral blood leukocytes, hematopoietic stem cells or fetal tissues (bone marrow, thymus and/or liver) in lymphopenic mice such as the NOD/SCID/Il2rg^−/−^ (NSG) mouse strain^15,16^. Recently, Wunderlich et al. reported that transgenic expression of human SCF, GM-CSF and IL-3 in NSG mice, named NSGS mice, improves hematopoietic engraftment, reconstitution and function^17,18^. However, because these lymphopenic mice do not express *Il2rg*, resulting in impaired signaling in lymphoid tissue inducer (LTi) cells, they do not have Peyer’s patches and lymphoid follicles in their intestine^19,20^. The lack of GALT in these mouse models represents a major limitation to understanding numerous human gastrointestinal diseases. Here, we aimed to bridge the gap by integrating human immune cells in a human intestinal tissue.

In our current model, we performed a serial time course following transplantation of HIOs under the kidney capsule to characterize the human immune cells that infiltrate the HIO lamina propria and epithelium. Mass cytometry and immunostaining confirmed the presence of GALT-associated B cells in cellular immune aggregates present in transplanted HIOs, resembling lymphoid follicles developing in human fetal intestine. Finally, we demonstrated that microbial exposure in the HIO lumen is required to induce the epithelial expression of glycoprotein 2 (GP2) at the cell surface of M cells, which consequently activate immune cells in the lamina propria. Altogether, these results demonstrate that crosstalk between the HIO epithelium and the immune cells induces the formation of lymphoid-like structures and M cell differentiation and function.

## Results

### Human immune cells integrate into transplanted HIOs

**Fig. 1:**
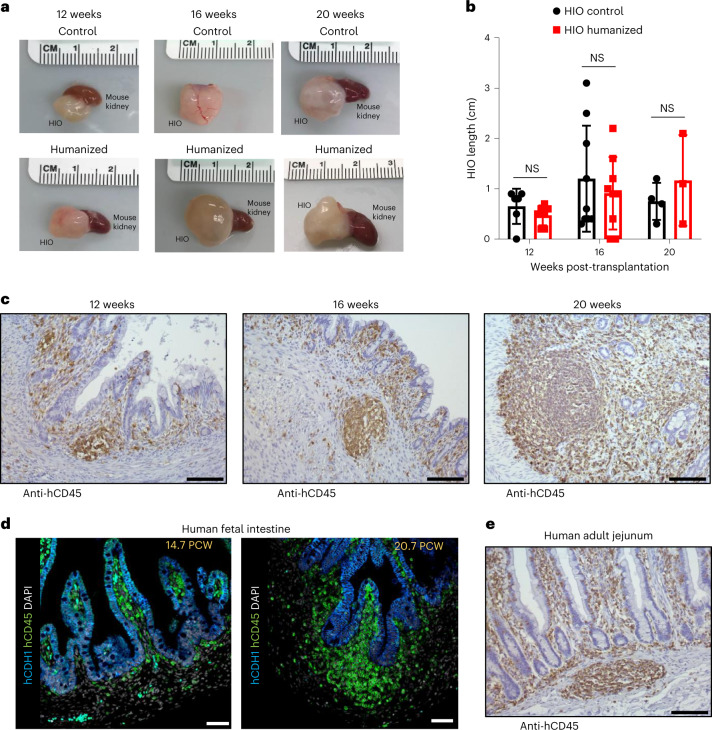
Integrating immune cells in HIO using immune system-humanized mouse model. **a**, Transplanted HIOs at 12, 16 and 20 weeks with mouse kidney seen underneath from control or humanized mice. **b**, Graph represents the length of HIOs from control (black circle) or humanized (red square) group at 12 weeks (*n* = 6 control and 7 humanized mice), 16 weeks (*n* = 9 control and 9 humanized mice) and 20 weeks (*n* = 4 control and 3 humanized mice) post-transplantation. Mean ± s.d. Multiple Mann–Whitney tests (two-sided); *P* = 0.1666 for 12 weeks, *P* = 0.7120 for 16 weeks and *P* = 0.714 for 20 weeks. NS, not significant. Graph representative of at least three independent experiments. **c**, Formalin-fixed paraffin-embedded sections of transplanted HIO at 12 (*n* = 5), 16 (*n* = 7) and 20 (*n* = 3) weeks stained by IHC with anti-human CD45 antibody. Scale bars, 100 μm. Representative of at least three independent experiments. **d**, Human fetal intestine at 14.7 and 20.7 PCW stained, by immunofluorescence, with anti-human CDH1 (E-cadherin) (blue), anti-human CD45 (green) antibodies and DAPI (white). Scale bars, 100 μm. Representative of two samples. **e**, Human adult jejunum stained by IHC with anti-human CD45 antibody. Scale bar,  100 μm. Representative of three samples.

**Extended Data Fig. 1:**
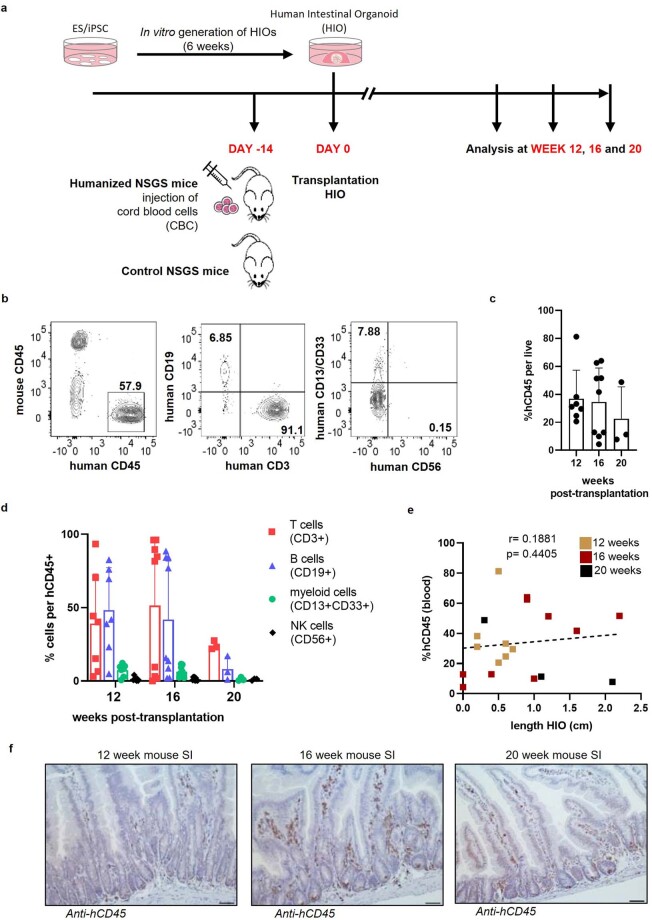
Validation of the humanized mouse model. **a**. Experimental workflow. Human intestinal organoids (HIOs) generated *in vitro* were then transplanted in humanized or control mice. At 12, 16 and 20 weeks post transplantation, HIOs and tissues of interest were collected for analysis. **b-d**. Flow cytometry analysis of peripheral blood from humanized mice. Contour plots represent the gating strategy of human immune cell lineages in humanized mice post HIO transplantation (**b**). Graph represents percentage of total hCD45+ cells (**c**) or, as indicated, immune cell subsets (**d**) in peripheral blood of humanized mice at 12 (n = 7), 16 (n = 9) and 20 (n = 3) weeks post HIO transplantation. Mean± standard deviation (SD). **e**. Graph represents relationship between length of HIO and percentage of blood hCD45 at different time points. Spearman correlation coefficient r = 0.1881 with *P* = 0.4405 (two-sided). (12 weeks *n* = 7; 16 weeks *n* = 9 and 20 weeks *n* = 3 mice) **f**. Humanized mouse small intestine at indicated time, were stained with anti-human CD45 by immunohistochemistry (IHC) and couterstained with hematoxylin. Scale bar represents 50 µm.

**Extended Data Fig. 2:**
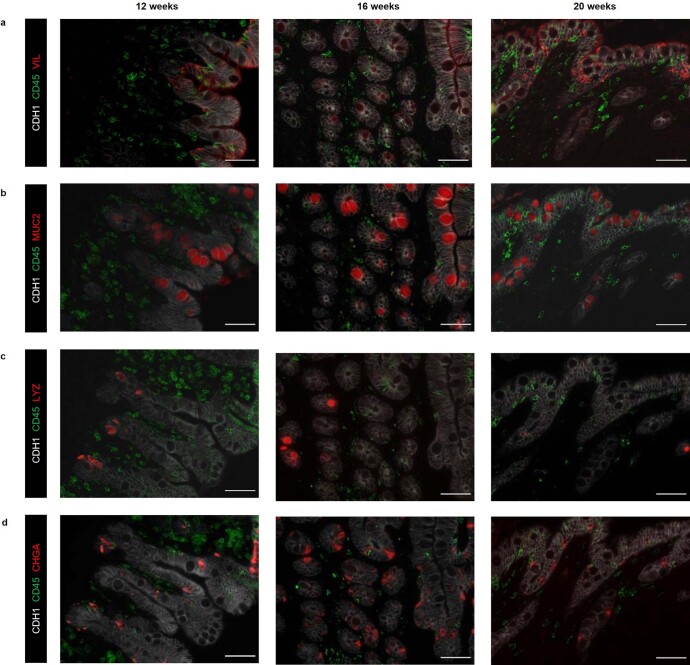
Intestinal cell lineage expression in HIO transplanted in humanized mice. **a-d**. Co-staining of intestinal markers (red), human CD45 (green) and human CDH1 (white) on sections of HIOs at 12, 16 and 20 weeks post transplantation in humanized mice. Images represent presence of enterocytes (VIL/Villin) (**a**), goblet cells (MUC2/Mucin2) (**b**), Paneth cells (LYZ/Lysozyme) (**c**) and enteroendocrine cells (CHGA/Chromogranin A) (**d**). Scale bar represents 100 µm. Representative of 2 independent experiments.

To bioengineer an HIO with immune cells, we transplanted PSC-derived HIOs under the kidney capsule of humanized NSGS mice (see workflow in Extended Data Fig. 1a). We collected and analyzed HIOs at 12, 16 and 20 weeks post-transplant. We confirmed the presence of human immune cells in the blood of humanized mice at each time point (Extended Data Fig. 1b–d) and found that HIOs successfully engrafted and grew to a similar size compared with those grown in nonhumanized control mice (Fig. 1a,b). However, we noticed that HIOs transplanted for periods of 16 and 20 weeks were more heterogeneous in size and this phenomenon may be due to an accumulation of mucus within the HIO lumen which cannot be drained out. Regardless, the effect is independent of the presence of immune cells as we observed the same heterogeneity in size at 16 and 20 weeks in control mice. In addition, there was no correlation between the size of HIO and the percentage of human immune cells in the peripheral blood of humanized mice (Extended Data Fig. 1e). By immunohistochemistry (IHC) staining, we demonstrated that human CD45^+^ cells had migrated to the mucosal layer and populated the lamina propria as well as the epithelium and formed cellular aggregates comparable to a human fetal or adult gut immune landscape (Fig. 1c–e). Notably, this did not occur within the humanized mouse small intestine (SI) (Extended Data Fig. 1f). As demonstrated in our previous study in immunodeficient mice^12^, HIOs engrafted and differentiated in humanized mice expressed all major intestinal cell lineages (Extended Data Fig. 2).

### Mass cytometry analysis reveals a GALT-like immune signature profile in HIO

**Fig. 2:**
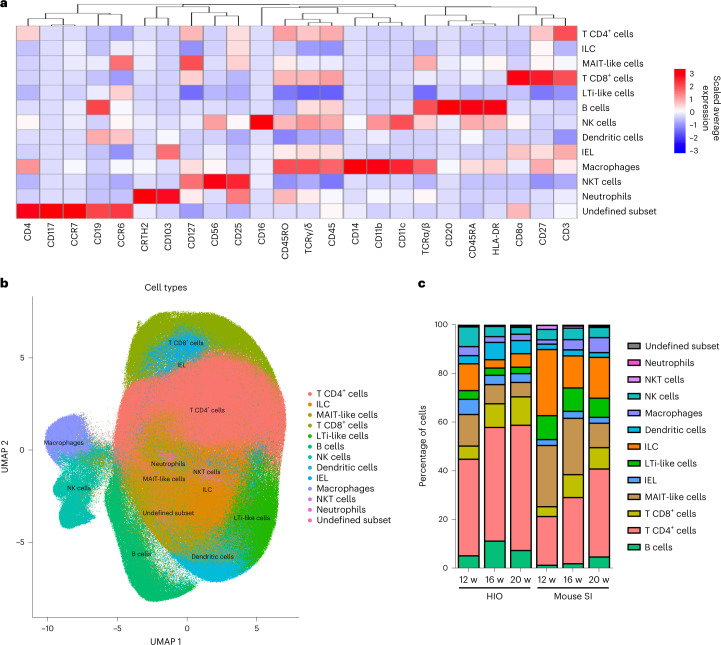
Immune profiling in HIOs and humanized mouse SI by mass cytometry. **a**, Heatmap illustrating the level of expression of each marker (*x* axis) for each cluster corresponding to their identified cell type (*y* axis). **b**, Visualization of high-dimensional data with UMAP overlaid with identified cell types. Heatmap (**a**) and UMAP (**b**) were generated with combined CyTOF dataset from transplanted HIO and humanized mouse SI at 12, 16 and 20 weeks post-transplantation. **c**, Stacked bar graph representing the frequencies of each cell type per tissue and time point. IEL, intraepithelial lymphocyte; LTi, lymphoid tissue inducer cell; ILC, innate immune cell; MAIT, mucosal-associated invariant T cell; NK, natural killer cell; NKT, natural killer T cell; w, weeks.

**Extended Data Fig. 3:**
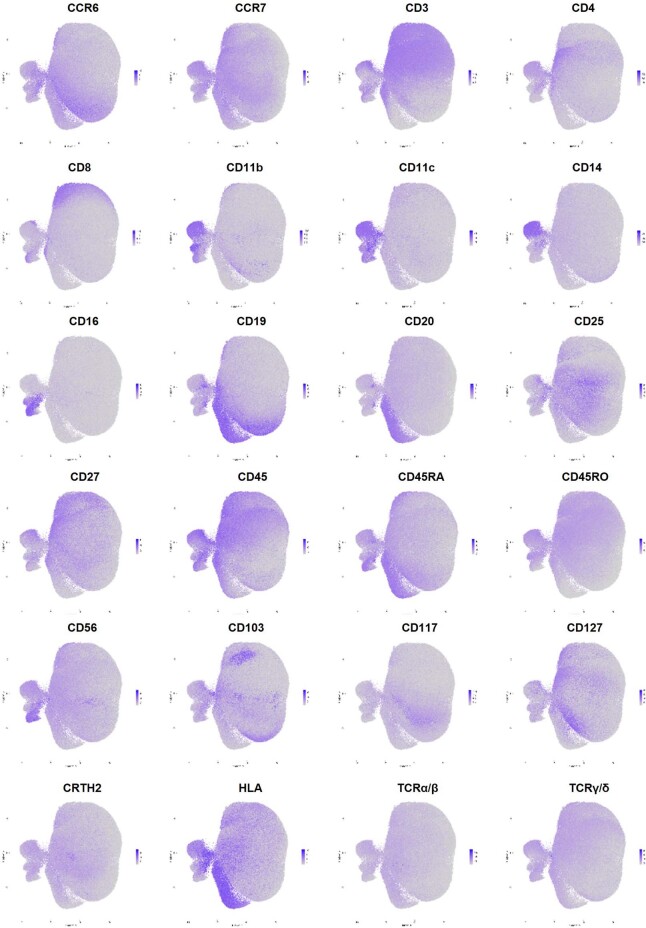
Profile expression of each marker in two-dimensional UMAP graph. UMAP graphs represent the expression of each marker across the samples.

**Extended Data Fig. 4:**
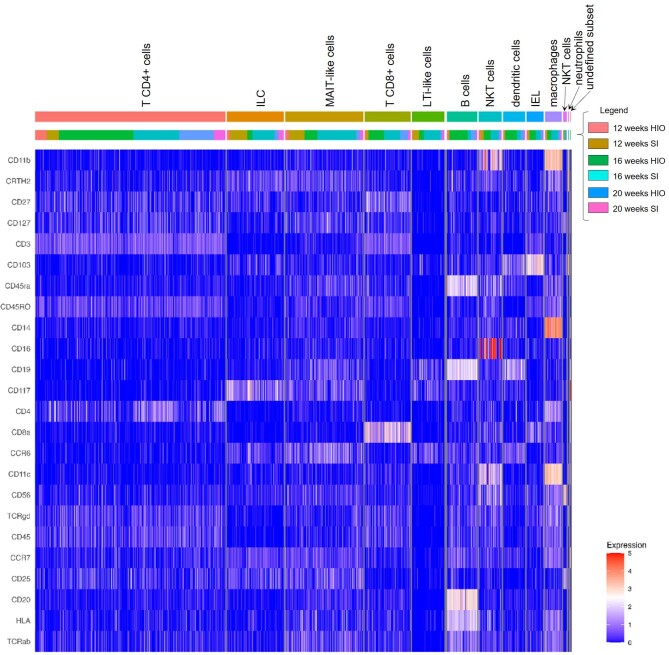
Expression of each markers at cellular level per cell types, tissues and time points. Heatmap graph represents the level of expression of each markers per cell. Each bar on the heatmap corresponds to a cell from a sample (HIO or mouse small intestine (SI) at 12, 16 or 20 weeks post transplantation). Top x axis corresponds to the cluster/identified cell type. Bottom x axis corresponds to the group: HIO or mouse small intestine (SI) at 12, 16 or 20 weeks (see color legend on top right corner).

**Extended Data Fig. 5:**
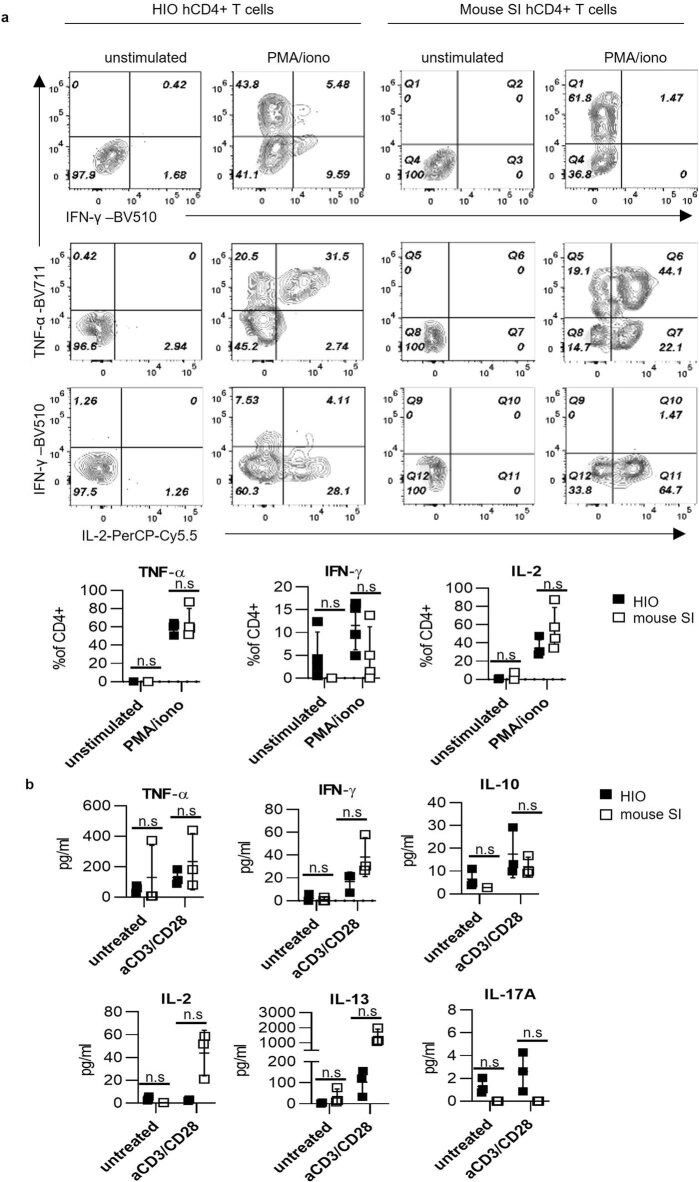
T cell cytokine profile from transplanted HIO is comparable to intestinal T cells from humanized mice. **a**. Contour plots illustrate the graphs (bottom) representing the expression of TNF-α, IFN-γ and IL-2 in unstimulated or PMA/ionomycin (PMA/iono)-treated human CD4+ T cells isolated from HIO (black square; n = 4 samples) or mouse small intestine (SI) (open square; n = 4 samples) from humanized mice at 16 weeks post transplantation. Mean±SD. Wilcoxon matched-pairs signed rank test (two-sided). Non-significant (n.s). **b**. Indicated cytokines were measured by multiplex (Luminex®) assay in supernatants of immune cells isolated from HIO (black square; n = 3 samples) or mouse small intestine (SI) (open square; n = 3 samples) of humanized mice at 16 weeks post transplantation and treated for 3 days with anti-CD3/CD28 antibody or untreated media. Mean±SD. Wilcoxon matched-pairs signed rank test (two-sided). Non-significant (n.s).

By mass cytometry, we next determined the immune signature in transplanted HIOs, and this was compared with the humanized mouse SI as an internal control. Using unsupervised clustering analysis, human CD45^+^ immune cells were classified and organized into 13 clusters as visualized in a heatmap and uniform manifold approximation and projection (UMAP) graph (Fig. 2a,b). Based on the combination and level of expression of markers on the heatmap and the expression patterns on individual UMAP graphs (Fig. 2a and Extended Data Figs. 3 and 4), we were able to define 12 immune cell types corresponding to the major immune cell lineages found in the intestine, for instance, T cells, B cells and innate lymphoid cells, as well as tissue-specific immune cells such as intraepithelial lymphocytes, mucosal-associated invariant T-like cells and LTi-like cells (Fig. 2a,b). To compare the immune profile between HIO and humanized mouse SI post HIO transplantation, we determined the proportion of each immune cell type in each group over time (Fig. 2c). Our results revealed a comparable immune cell composition between the groups, except that B cells and CD4^+^ T cells were present in relatively higher numbers in HIOs compared with humanized mouse SI with increasing abundance from 12 to 16 weeks. However, further increases were not seen in the immune profile at 20 weeks post HIO transplantation compared with the profile at 16 weeks. There was no inflammation associated with the increase in CD4^+^ T cells, as demonstrated by a similar cytokine profile in cells isolated from HIOs or humanized mouse SI (Extended Data Fig. 5a,b). Notably, in the SI, B cells are mostly found in GALT structures such as lymphoid follicles or Peyer’s patches which also contained CD4^+^ T cells. Our humanized mice lack IL-2Rγ, indicating that they lack intestinal lymphoid follicles and Peyer’s patches, resulting in fewer intestinal T and B cells which correlates with our mass cytometry results. Taken together, our findings suggest that the augmentation of B and T cell frequencies observed in HIOs could come from GALT structures.

### Immune aggregates in the developing HIO are lymphoid follicle-like structures

**Fig. 3:**
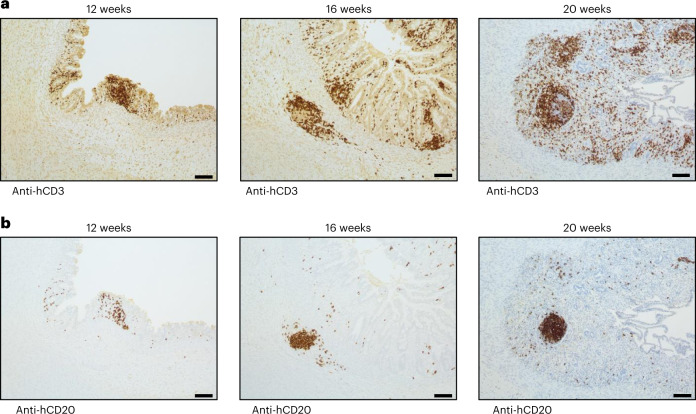
Immune cellular aggregates in transplanted HIO contain T and B cells. **a**,**b**, Presence of human T cells (anti-hCD3) (**a**) and human B cells (anti-CD20) (**b**) in HIO at 12 (*n* = 5), 16 (*n* = 7) and 20 (*n* =3) weeks post-transplantation. Scale bar, 100 μm. Representative of three independent experiments.

**Extended Data Fig. 6:**
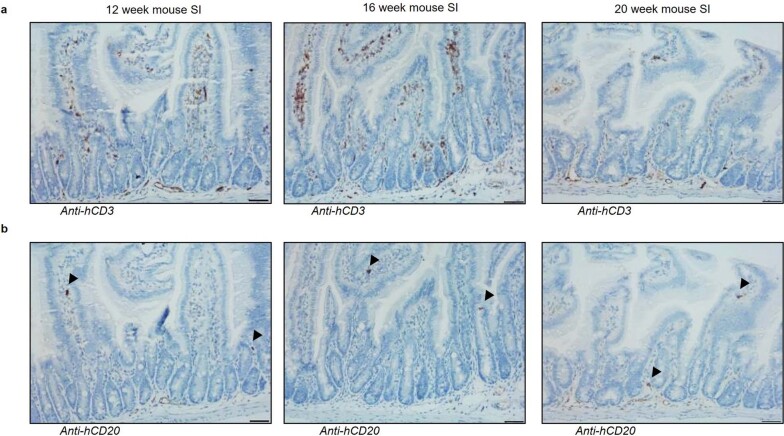
Presence of T cells with few scattered B cells in humanized mouse small intestine. Humanized mouse small intestine at 12, 16 and 20 weeks post HIO transplantation, were stained with anti-human CD3 (T cells) (**a**) or anti-human CD20 (B cells) (**b**) by immunohistochemistry(IHC) and couterstained with hematoxylin. In panel (**b**) black arrowheads indicate presence of B cells in humanized mouse small intestine. Scale bar represents 50 µm. Representative of 3 independent experiments.

Lymphoid follicles are well-organized structures formed by aggregation of B cells surrounded by T cells. IHC staining with anti-human CD3 and CD20 antibodies revealed that aggregates found in HIOs contained T and B cells, respectively, at all time points (Fig. 3a,b). Surprisingly, in contrast to 12-week-old transplanted HIOs, a distinct cellular zonation of T and B cell populations appeared in 16- as well as 20-week-old HIOs resembling a lymphoid follicle-like structure (Fig. 3a,b). However, we did not observe any aggregates of T and B cells in the humanized mouse SI at any time point (Extended Data Fig. 6a,b). This result suggests that HIOs promote and influence the formation and maturation of lymphoid follicular structures over the course of their development.

### Temporal and spatial development of lymphoid-like structures in transplanted HIOs correlate to human lymphoid follicle development

**Fig. 4:**
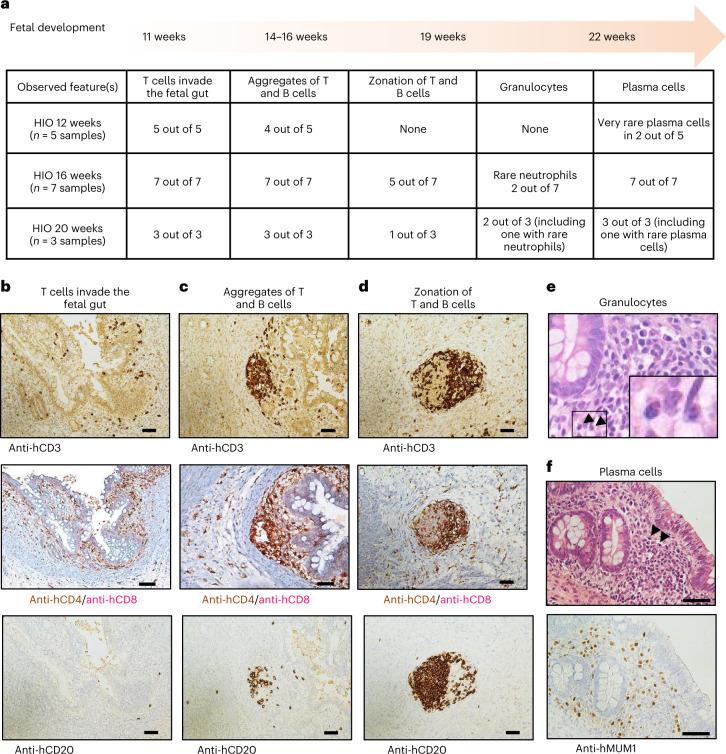
Lymphoid-like structures in transplanted HIOs correlate to lymphoid development in fetal gut. **a**, Prevalence of immune developmental features observed in HIO. Representative of two independent experiments^21,22^. **b**–**f**, Images illustrating features observed in HIO that resemble immune cell development described in fetal gut. **b**–**d**, HIO sections stained with anti-human CD3 (top) to demonstrate the presence of T cells, anti-human CD4/CD8 (middle) to distinguish T helper cells versus T cytotoxic cells and anti-human CD20 (bottom) to highlight the presence of B cells. **e**, Hematoxylin and eosin (H&E) staining indicating the presence of neutrophils (black arrowheads) observed in late developing HIOs only. **f**, H&E staining highlight the presence of plasma cells (black arrowheads, top) then confirmed with anti-human MUM1 IHC staining (bottom). Scale bars, 50 μm. Representative of two independent experiments.

**Extended Data Fig. 7:**
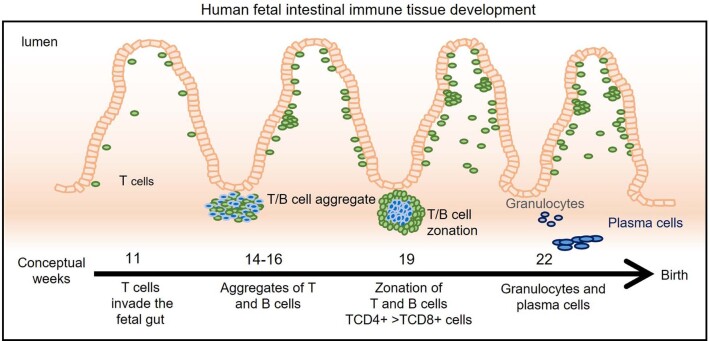
Mechanism of human lymphoid follicle development. Schema summarizes the cellular mechanism of lymphoid follicle formation during fetal gut development described by Spencer et al. and Braegger et al. (see references^21,22^). Briefly, around 11 post conceptual weeks (PCW), T cells start to invade the gut followed by B cells. Around 14 to 16 PCW, T and B cells form aggregates and later mature into lymphoid follicles, at 19 PCW. Plasma cells as well as granulocytes were observed in fetal intestine at 22 PCW.

Because the developing HIO is fetal in nature^11^ and the lumen lacks exposure to antigens and microbiota, we compared the immune aggregates developing in HIO with what has been observed previously in the developing human fetal gut. Lymphoid follicles have been reported to develop in the human fetal gut starting at the second trimester of gestation^21,22^. In Extended Data Fig. 7, we summarized these findings on the developing human fetal gut immune tissue and used them as a reference to compare with the features observed in transplanted HIOs. Starting at 12 weeks post HIO transplant, we found in the lamina propria as well as the epithelium the presence of T cells (mostly CD4^+^ cells) with few scattered CD20^+^ B cells, pointing out a similarity with the cellular composition of a fetal intestine at 11 post conceptual weeks (PCW)^21,22^ (Fig. 4a,b). Additionally, in some areas, we observed the colocalization of T and B cells which resemble the aggregates described in the fetal intestine at 14–16 PCW (refs. ^21,22^) (Fig. 4a,c). Notably, the cellular zonation of T and B cells observed in the fetal intestine at 19 PCW (refs. ^21,22^) was found in 16- and 20-week HIOs, where T cell zones are represented with high proportions of CD4^+^ T cells and low CD8^+^ T cells (Fig. 4a,d). As described in human fetal intestine^21,22^, we also observed the presence of plasma cells and few neutrophils in both 16- and 20-week HIOs, demonstrating a more mature and complex cellular composition over the course of HIO differentiation (Fig. 4a,e,f). These data confirm that the HIO environment expresses unique signals to influence the maturation of the immune system as well as the formation of a lymphoid tissue.

### M cells are induced in transplanted HIOs after microbial exposure

**Extended Data Fig. 8:**
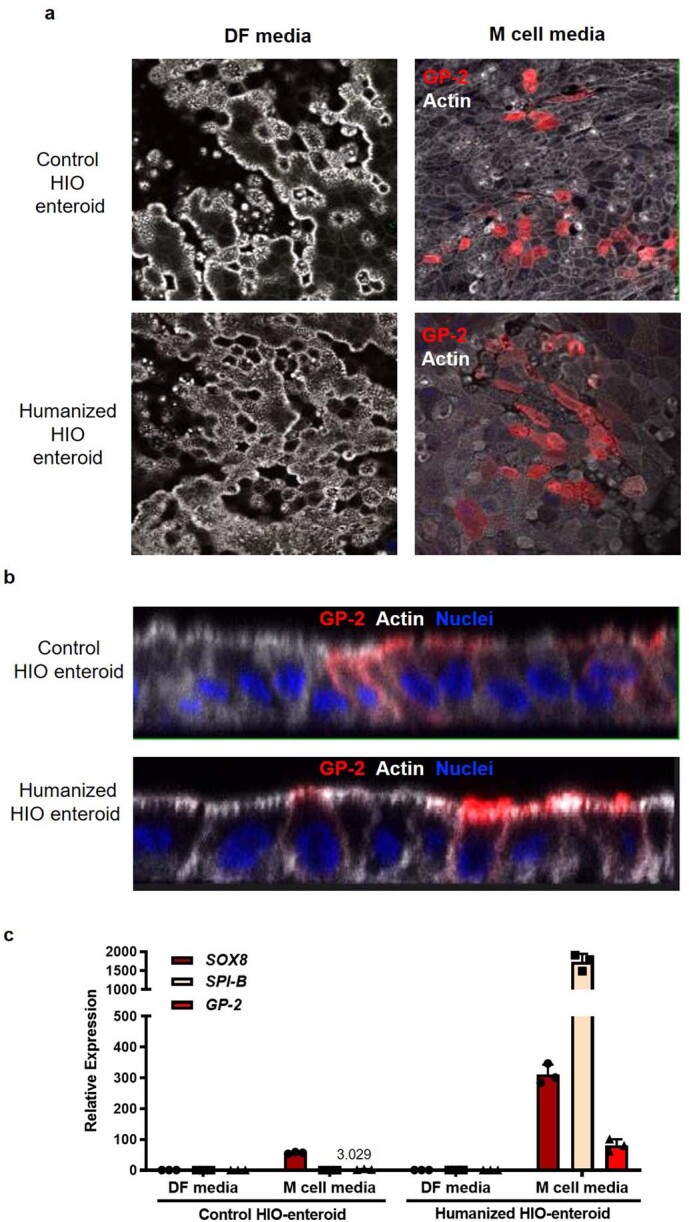
HIO-derived enteroids express M cell *in vitro*. **a**. Enteroid monolayers were grown to confluence, differentiated for 5 days (with DF or M cell media), immunostained with glycoprotein 2 (GP-2) and actin and imaged by confocal microscopy. **b**. Enteroid monolayers grown in M cell media and stained with GP-2 and imaged by confocal microscopy. **c**. qPCR for M cell specific transcription factors SOX8 and SPI-B as well as mature M cell marker GP2. n = 3 (technical triplicate). Mean±SEM. Representative of 1 independent experiment.

M cells are expressed in the epithelium overlying follicles and play a key role in immune responses by transporting antigens from the lumen to the lamina propria^2^. Crosstalk between lymphocytes and epithelial cells is known to promote M cell differentiation^23^. By histology, we were not able to detect GP2, a marker for M cells, at baseline in HIOs post-transplantation. However, in organoids derived from transplanted HIO epithelium, called enteroids, we found that GP2 was induced in vitro and expressed at a high level in enteroids derived from HIOs transplanted in humanized mice compared with control mice (Extended Data Fig. 8).

**Fig. 5:**
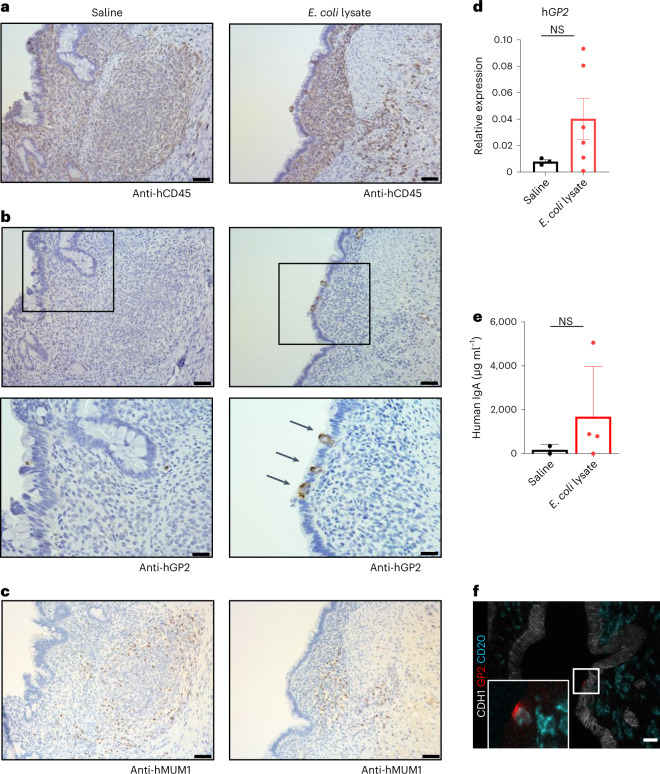
M cells are induced after microbial exposure in transplanted HIOs. **a–c**, 16-week HIO sections at 72 h post injection with saline (*n* = 3) (left) or *E. coli* lysate (*n* = 4) (right) stained with anti-human CD45 (**a**), anti-human GP2 (**b**) and anti-MUM1 (**c**). Arrows indicate M cells positive for human GP2. Scale bars represent 100 µm, except **b** (bottom) human GP2 scale bars represent 50 µm. Representative of two independent experiments. **d**, Graph represents level of human *GP2* gene expression evaluated by qPCR in 16-week HIO at 72 h post injection with saline (*n* = 3) or *E. coli* lysate (*n* = 6). Human *GP2* gene expression is normalized to human *GAPDH* gene. Mean ± s.d. Mann–Whitney test (two-sided); *P* = 0.1667. NS, not significant. **e**, Level of human IgA measured by ELISA in mucus from 16-week HIO 72 h after being injected with saline (*n* = 2) or *E. coli* lysate (*n* = 4). Mean ± s.d. Mann–Whitney tests (two-sided); *P* = 0.533. NS, not significant. **f**, Immunofluorescence staining with anti-human CDH1 (white), anti-human GP2 (red) and anti-human CD20 (blue) in 16-week HIOs injected with *E. coli* lysate (*n* = 4) for 72 h. Scale bar, 50 µm. Representative of two independent experiments.

GP2 is a transcytotic receptor and binds FimH, an adhesin molecule expressed by *Escherichia coli*. It was reported that the number of M cells increased after bacterial challenge^24^. Therefore, we aimed to determine if bacterial exposure could induce M cells in HIOs at 16 weeks post-transplantation. Indeed, we found that 72 h after administration of *E. coli* lysate in HIO lumen, GP2 was detected on the cell surface of epithelial cells overlying immune cells, indicating the presence of M cells (Fig. 5a,b). In contrast, transplanted HIOs injected with saline only demonstrated background expression, probably induced by antigens present in the environment at the time of injection. In line with our histology results, we confirmed by quantitative PCR (qPCR) the induction of *GP2* expression in transplanted HIOs after *E. coli* exposure compared with the saline group (Fig. 5d). M cell-mediated translocation of luminal antigens is required to initiate the production of IgA antibodies^2^. To determine whether M cells were functional and able to translocate luminal antigens to activate immune cells, we measured the level of IgA secreted in the mucus of transplanted HIOs in response to *E. coli* lysate injection. Even though plasma cells were present in both groups, unlike in the saline-treated group, we found that IgA antibodies were present at a high level in the mucus of HIOs exposed to *E. coli* lysate (Fig. 5c,e). This result indicates that M cells are functional in translocating antigens to activate immune cells, which subsequently respond to the microbial exposure by activating plasma cells to produce IgA antibodies. We then confirmed in the *E. coli*-treated group a colocalization of M cells and B cells by immunofluorescence, indicating a crosstalk between epithelial and immune cells (Fig. 5f).

## Discussion

Here, we report a next-generation in vivo model of HIOs with functional human immune tissue. Using this model, we demonstrate that immune cells temporally infiltrate and populate the HIO in the mucosa similarly to the immune landscape in the developing human gut. Mass cytometry data and immunostaining demonstrated the formation of GALT-like structures during HIO development, and their formation correlated temporally and spatially with human intestinal immune tissue development. Because M cells are present in the epithelium overlaying the GALT, we confirmed their presence and validated their function by exposing the HIO lumen to microbial components.

Intestinal immune tissue contains diverse immune cell types to protect the mucosal barrier against invaders^25^. The human fetal gut has a diverse, functional immune system even without being exposed to antigens^26–30^. Our mass cytometry analysis indicated that the immune cell composition was diverse and increased in cell number as the transplanted HIOs developed, similar to the human fetal gut. Our results correlate with recent mass cytometry and transcriptomic data showing an increase in immune cell number and cell subsets, in particular CD4^+^ T cells, during human fetal intestinal development^29,30^. During HIO development, we found by mass cytometry an increase in CD4^+^ T and B cell frequencies and we confirmed by histology that these cell types together formed aggregates that later developed into lymphoid follicles. These features were not identified in the intestine of humanized mice, confirming reports describing the absence of lymphoid follicles in this model^19,20^.

**Extended Data Fig. 9:**
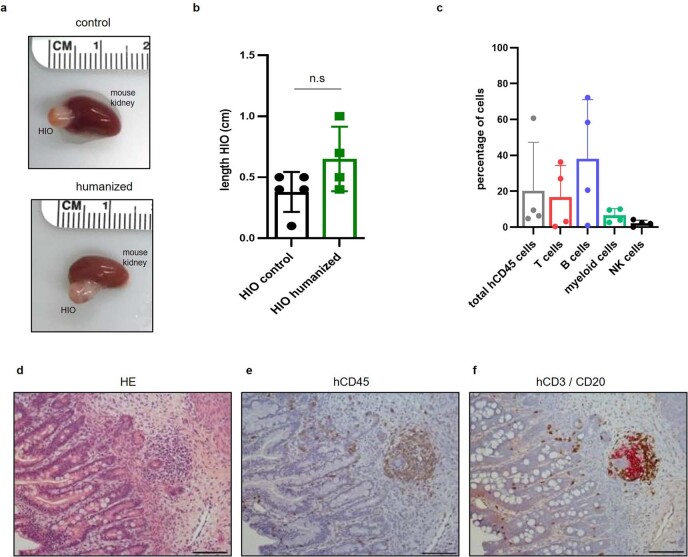
HIO microenvironment orchestrates GALT formation independently of the timing of hematopoietic reconstitution. 10 weeks after UCB engraftment, HIOs were transplanted and grown for 12 weeks. **a**. Transplanted HIO at 12 weeks with mouse kidney seen underneath from control or humanized mice. **b**. Graph represents the length of HIOs from control (n = 5) (black dot) or humanized (n = 4) (green square) group. Mean± standard deviation (SD). Mann-Whitney tests (two-sided); p = 0.1905. **c**. Graph represents immunophenotyping of peripheral blood from humanized mice at 12 weeks post HIO transplantation (or 22 weeks post UCB engraftment) (n = 4). Mean± standard deviation (SD). **d-e**.Formalin-fixed paraffin-embedded (FFPE) sections of transplanted HIO at 12 weeks H&E-stained (**d**) or stained by IHC with anti-human CD45 (**e**) and anti-human CD3 (brown) and CD20 (red) antibodies (**f**). Scale bar represents 100 μm.

The timing of humanization and transplantation did not affect the timing of GALT structure formation. In a preliminary study in which HIO transplantation was delayed until the immune system was fully reconstituted, histology and flow cytometry studies indicated that the development of immune cellular aggregates composed of T and B cells was similar to that of early HIO transplantation (Extended Data Fig. 9). These observations indicate that immune precursors were present even after the immune system was reconstituted. Taken together, our results suggest that the developing HIO and cells seeding the lamina propria influence and frame the formation of immune aggregates by expressing specific signals to attract and activate immune precursors as well as serving as a foundation for subsequent lymphoid tissue development.

**Extended Data Fig. 10:**
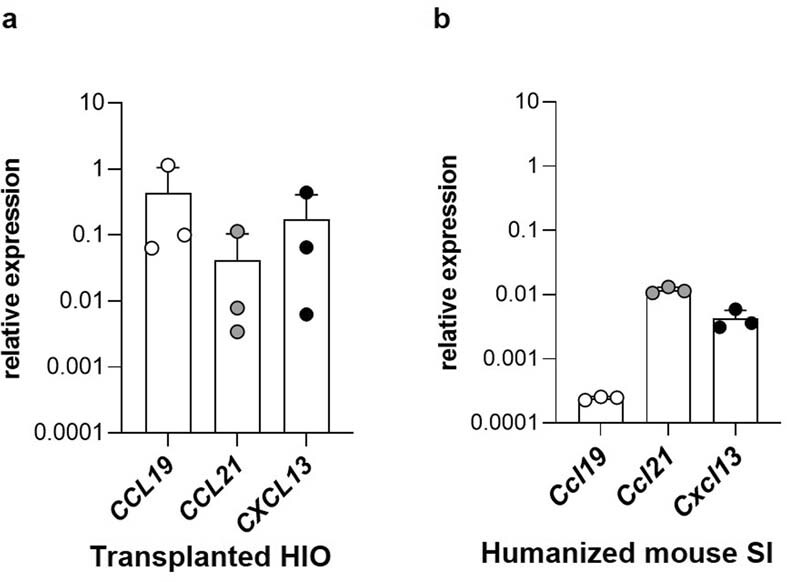
GALT-associated chemokines are expressed in HIO at 16 weeks. Graphs represent gene expression of indicated chemokines in transplanted HIO (**a**) or humanized mouse small intestine (SI) (**b**) at 16 weeks. n = 3 HIO and n = 3 humanized mouse SI. Gene expression is normalized to housekeeping gene (GAPDH). Mean± standard deviation (SD).

Recently, transcriptomic analysis of human fetal intestine has identified subsets of fibroblasts in GALT formation that correspond to lymphoid tissue organizer cells^30^. These cells increased in number with fetal development and interacted with diverse immune cell populations, including LTi cells via the expression of CCL19, CCL21 and CXCL13 (ref. ^30^). In our model, preliminary data indicated that *CCL19*, *CCL21* and *CXCL13* were expressed in HIOs at 16 weeks post-transplantation, whereas their mouse gene orthologs were decreased by at least tenfold (Extended Data Fig. 10a,b). These results indicate that, in contrast to the humanized mouse SI, the HIOs express unique microenvironment signals that support the formation of GALT-like structures. In line with this, our model could be used to dissect the mechanism of interaction between stromal lymphoid tissue organizer cells and LTi cells and to identify key pathways in the development of GALT in humans.

GALT is a site of adaptive immune responses and influences the epithelial cells overlaying the follicles to promote the generation of M cells that transport luminal antigens to activate immune cells^2^. GP2 is a glycoprotein expressed on the apical side of M cells and translocates luminal antigens to the immune cells in the lamina propria. Even though transplanted HIOs expressed the *GP2* gene, we were not able to detect the protein by IHC likely due to the lack of microbial antigens in the lumen, similar to a fetal intestine^11^. Of note, nonhumanized HIOs exposed to microbial antigens in other models being developed in the laboratory also do not express GP2 protein (data not shown). Using our current model, we demonstrated that exposing the HIO lumen to *E. coli* lysate induced M cells, characterized by GP2 expression as well as activation of an immune response indicated by the presence of IgA antibodies. After being produced by plasma cells in the lamina propria, IgA antibodies bind to polymeric immunoglobulin receptors (pIgR) expressed on the basal side of epithelial cells and, by transcytosis, epithelial cells then secrete IgA antibodies in the intestinal lumen^31^. Because we detected the presence IgA antibodies in the mucus, our findings suggest that epithelial cells in HIOs express pIgR and transport antibodies to the lumen.

Here, we have demonstrated that our in vivo HIO model with GALT-like structures and M cells provides opportunities for studies on food allergies, intestinal infectious diseases and the development of mucosal vaccines.

## Methods

### Animals

Immunodeficient NOD/Scid Il2rg null Tg (hIL3, hGM-CSF and hSCF) (NSGS; The Jackson Laboratory no. 013062 (ref. ^17^)) mice were bred and housed in the animal facility at the Cincinnati Children’s Hospital Medical Center (CCHMC). All experiments were performed with the approval of the Institutional Animal Care and Use Committee of CCHMC (IACUC protocol no. 2018-0092).

### Human intestinal tissue

Normal, de-identified developing human fetal tissues from elective abortions were obtained from the University of Washington, Laboratory of Developmental Biology, and all work was approved by the University of Washington and the University of Michigan Institutional Review Boards (IRBs).

Normal adult human jejunum was obtained from patients undergoing bariatric procedures between the ages of 16 and 25 yr. Informed consent or assent was obtained from all patients and/or parents/legal guardians as appropriate. Human tissue collection was performed with the previous approval of CCHMC’s IRB.

### HIOs

HIOs were generated and maintained as previously described^12,32,33^.

Briefly, human H1 embryonic stem cells (WA-01; WiCell) (passage number 40–55), obtained from the Pluripotent Stem Cell Facility in our institute, were grown in feeder-free conditions in mTESR1 medium (Stem Cell Technologies). For induction of definitive endoderm, cells were split with Accutase (Invitrogen) and plated at a density between 70,000 and 100,000 cells per well in a Matrigel-coated 24-well plate. Once the cells reached 80–95% confluency, they were treated with 100 ng ml^−1^ Activin A for 3 d as previously described^34^. Definitive endoderm was then treated for 4 d with hindgut induction medium containing 500 ng ml^−1^ FGF4 (R&D) and 3 μM Chiron 99021 (Tocris) to induce formation of mid-hindgut spheroids. Spheroids were then plated in Matrigel (Corning) and maintained in intestinal growth medium supplemented with 100 ng ml^−1^ EGF (R&D) to generate HIOs. Medium was changed twice weekly and HIOs were replated in fresh Matrigel at 14 d.

### Human umbilical cord blood cell engraftment

The Translational Trials Development Support Laboratory of CCHMC collected and distributed the umbilical cord blood units according to an IRB-approved protocol (protocol no. 02-3-4x). Mice were humanized as described previously^35^. Briefly, whole cord blood was subjected to hetastarch-induced aggregation of red blood cells. Cord blood cells (CBCs) were isolated, washed and viably frozen for future use. Thawed CBCs were resuspended in IMDM medium with 3% FBS and antibiotics and diluted to 23.3–28.3 × 10^6^ CBCs per ml. OKT3 antibody was spiked into the cell solution at a concentration of 1 µg per 1 million cells to prevent graft-versus-host disease. Immunodeficient NSGS mice at 6–8 weeks old were conditioned by receiving a dose of busulfan (30 mg kg^−1^ by intraperitoneal injection) 24 h before intravenous injection of 7–8.5 × 10^6^ CBCs in 300 µl. Busulfan is a chemotherapy drug that partially depletes cells from the mouse bone marrow and allows the human hematopoietic stem cells to engraft. As a preventative measure against possible systemic infections, mice were fed with doxycycline chow (0.0625%, Purina) for 2 weeks before and after human cell engraftment.

### Transplantation of HIOs

As previously described, a single HIO, matured in vitro for 28 d, was removed from Matrigel and then transplanted under the kidney capsule^12^. Briefly, the mice were anesthetized with 2% inhaled isoflurane (Butler Schein) and 2.5–3 l min^−1^ oxygen. The left side of the mouse was then prepped in sterile fashion with isopropyl alcohol and providine-iodine. A small left-posterior subcostal incision was made to expose the kidney. A subcapsular pocket was created in the kidney capsule and the HIO was then placed into the pocket. The kidney was then returned to the peritoneal cavity and the mice were given an intraperitoneal flush with 2–3 ml of piperacillin/tazobactam (100 mg kg^−1^; Pfizer) to help prevent bacterial infection. The skin was closed in a double layer. For pain control, mice were then given a subcutaneous injection with buprenorphine (0.05 mg kg^−1^; Midwest Veterinary Supply) or carprofen (4 mg kg^−1^; Midwest Veterinary Supply) and were monitored for the next 48 h following surgery. Additional injections of pain medication were given if needed. At 12 and 16 weeks following engraftment, the mice were then euthanized and the tissues were collected and analyzed.

#### *E. coli* lysate preparation and injection

Grown in LB medium overnight, *E. coli* suspension was then washed three times with PBS and centrifuged at 800 *g* for 5 min. Bacteria were resuspended in saline solution at ~10^7^ colony-forming units per ml and centrifuged at 10,000 *g* for 5 min at 4 °C. Bacteria were then lysed by freeze–thaw cycles repeated four times.

At 16 weeks post HIO transplantation, the mice were anesthetized and a small left-posterior subcostal incision was made to expose the transplanted HIO. Then, 50–100 µl of *E. coli* lysate was injected with a 0.5-ml insulin syringe in the HIO lumen.

The HIO was then returned to the peritoneal cavity and the skin was closed in a double layer. For pain control, mice were then given a subcutaneous injection with carprofen (4 mg kg^−1^; Midwest Veterinary Supply) and were monitored for the next 48 h following surgery. Additional injections of pain medication were given if needed. At 72 h post injection, the mice were euthanized and the tissues were collected and analyzed.

### Flow cytometry

To confirm the expression of human immune cells from CD34^+^ engraftment in mice, retro-orbital bleeding was performed 8–10 weeks after the engraftment and a day before each tissue collection. Approximately 50 μl of whole blood was collected in a BD Microtainer Dipotassium/EDTA-coated tube and then lysed in 5 ml of red blood cell lysis buffer (155 mM NH_4_Cl, 12 mM NaHCO_3_ and 0.1 mM EDTA pH 8.0; diluted in dH_2_O), for 5 min at room temperature. An isovolume of PBS was added and tubes were centrifuged at 300 *g* for 5 min. The pellet was washed in PBS, centrifuged and resuspended in FACS buffer (0.5% BSA, 2 mM EDTA in PBS 1×). Cells were stained for 30 min on ice with the following combination of antibodies: FITC-conjugated anti-human CD45, PE-Cy5-conjugated anti-mouse CD45, BV650-conjugated anti-human CD19, PE-Cy7-conjugated anti-human CD3, BV421-conjugated anti-human CD56, PE-conjugated anti-human CD13, PE-conjugated anti-human CD33 and Zombie NIR fixable viability kit to exclude dead cells (Supplementary Table). All antibodies were used at 1:200 dilution except Zombie NIR which was used at 1:2,000 dilution. Samples were washed twice and resuspended in FACS buffer. The samples were then recorded on an LSR Fortessa instrument (BD Biosciences) and the data were analyzed with FlowJo software (TreeStar).

### HIO and SI dissociation and cell preparation

One-third of the HIO was used for cell dissociation. The cell dissociation protocol for the HIOs was modified from Weigmann’s protocol for mouse colon cell dissociation^36^. Briefly, HIOs were cut into small pieces and incubated under slow rotation for 20 min at 37 °C in 5 ml of predigestion solution containing EDTA and dithiothreitol in HBSS. The epithelial cell suspension was filtered through a 100-μm cell strainer, washed with cold PBS and kept on ice until pooled with the lamina propria-isolated cells. The remaining pieces of HIO were minced and placed in a new tube with 5 ml of digestion solution containing collagenase D (Roche), DNAse I (Roche) and dispase II (Roche) in PBS and incubated for 15 min at 37 °C under slow agitation. The lamina propria cell suspension was passed through a 70-μm cell strainer, washed with cold PBS and re-incubated in 5 ml of digestion solution for 15 min at 37 °C under slow agitation. This step was repeated one more time. Epithelial and lamina propria cells were combined in one tube and spun down at 450 *g* for 5 min.

The method to isolate mononuclear cells from the humanized mouse gut was adapted from the protocol reported by Lee et al.^37^. Briefly, 10–12 cm of proximal SI was collected, longitudinally cut open and washed in HBSS to remove any debris. The SI was incubated in HBSS containing 5 mM EDTA on ice for 5 min and vortexed at medium intensity, for a total of four incubations. The epithelium was then collected in a separate tube. After digestion of the tissue at 37 °C for 30 min with DNAse I (Roche) and Collagenase A (Roche), the homogenate was filtered and centrifuged at 450 *g* for 5 min.

Both HIO and SI cell suspensions were individually resuspended in 44% Percoll (GE), loaded on a 67% Percoll layer and centrifuged at 650 *g* without brake for 30 min at room temperature. The cell layer at the interface of the two gradients was collected, washed, counted and used for in vitro assays, or stained for immune markers and analyzed by mass cytometry.

### Mass cytometry staining and data analysis

#### Mass cytometry staining

Antibodies used in this panel were purchased from Fluidigm except anti-human CD45RO antibody which was purchased from BioLegend and was labeled with Maxpar X8 Antibody Labeling Kit (Fluidigm) according to the manufacturer’s instructions. All the reagents used in the following protocol were purchased from Fluidigm and all incubations were done at room temperature. Samples were first stained for 5 min with Cell-ID Cisplatin at a final concentration of 5 μM in Maxpar PBS and then washed in 5 volume of Maxpar Cell Staining Buffer. Before being stained for cell surface markers, cells were fixed in Fix I Buffer for 10 min, washed twice with Maxpar Barcode Perm Buffer and barcoded with Cell-ID 20-Plex Pd Barcoding Kit for 30 min. Samples were pooled and then stained for 30 min with a cocktail of 25 antibodies in a final volume of 100 μl of Maxpar Cell Staining Buffer (antibody dilution 1:200) (Supplementary Table). After cell surface staining, samples were washed, fixed in 1.6% paraformaldehyde (PFA) (EMS) for 10 min and, finally, incubated in 1 ml of intercalator solution at a final concentration of 125 nM. The samples were kept cold and shipped in intercalator solution to the Cytometry Research Core Facility at the University of Rochester, NY. The samples were washed and resuspended in Maxpar water before being acquired using a cytometry by time of flight (CyTOF)/mass cytometry Helios instrument (Fluidigm). Data were then debarcoded using Fluidigm debarcoder software (available online).

#### Bioinformatics analysis

CyTOF datasets from transplanted HIO- and humanized mouse SI-isolated immune cells were generated using mass cytometry (Fluidigm) variation at three time points, 12 weeks, 16 weeks and 20 weeks. Datasets at aforementioned time points were generated in two batches for each tissue at 12 and 16 weeks post-transplantation with a total of *n* = 7 samples and *n* = 10 samples, respectively, and in one batch for each tissue at 20 weeks post-transplantation. Combining two batches, ~1.1 million cells were analyzed, amounting to 63,076 cells in 12-week HIO, 350,132 cells in 16-week HIO, 148,274 cells in 20-week HIO, 137,887 cells in 12-week SI, 368,893 cells in 16-week SI and 71,569 cells in 20-week SI.

Raw .fcs files for each sample were read and analyzed using *read.flowSet* function from the flowCore R (v.3.6.3) package. The .fcs files stored in *flow.set* objects were than normalized using inverse hyperbolic sine (*Arcsinh*) transformation (https://support.cytobank.org/hc/en-us/articles/206148057-About-the-Arcsinh-transform) using a cofactor value of 5. Normalized reads from the .fcs files were then used to create a Seurat object using *CreateSeuratObject* function from Seurat (v.3.0.2), a single-cell analysis package in R.

For each Seurat object, the following steps were carried out: variable features set to surface markers, scaling, dimension reduction and clustering. Dimension reduction was applied in both linear (principal components) and nonlinear (UMAP) approaches to obtain respective components. Clustering was performed using a *k*-nearest neighbor graph of cells based on marker abundance similarity. This *k*-nearest neighbor graph was finally partitioned into clusters based on highly interconnected colonies of cells.

In the end, all the processed Seurat objects for each sample from two batches were integrated together using Seurat’s reciprocal principal component analysis as the numbers of cells were large (~1.1 million). Briefly, each dataset was projected into principal component space of other datasets to learn the anchors (cell pairs) based on the mutual nearest neighbor graph requisite.

Dimension reduction plots were generated using *DimPlot* function and heatmaps were generated using the *DoHeatmap* function in Seurat.

Example datasets and analysis code have been deposited to GitHub: https://github.com/praneet1988/Analyze_CyTOF_Using_Seurat.

### Measurement of cytokines from HIO- and mouse SI-derived immune cells

#### Detection of cytokines by flow cytometry

Immune cells isolated from HIO or mouse SI were stimulated for 4 h with 1:500 Cell Activation Cocktail (BioLegend) containing, according to the manufacturer, an optimized concentration of phorbol 12-myristate-13-acetate (PMA) and ionomycin. After an hour of stimulation, cells were then incubated with 1:1,000 Brefeldin A Solution (Biolegend) to block the secretion of cytokines. Finally, the cells were stained with anti-human CD3 and anti-human CD4 antibodies (Supplementary Table), fixed with Cytofix (BD Biosciences) and then permeabilized with CytoPerm (BD Biosciences) overnight at 4 °C. The following day anti-IFNγ, anti-TNFα and anti-IL-2 antibodies (Supplementary Table) were added to the cells for at least 1 h at room temperature. Samples were washed twice and resuspended in FACS buffer. The samples were then recorded on an Aurora instrument (Cytek) and the data were analyzed with FlowJo software (TreeStar).

#### Detection of cytokines by Milliplex assay

Indicated cytokines were measured in supernatants from immune cells (cell density 10^6^ per ml) isolated from HIO or mouse SI stimulated for 3 d with a cocktail of anti-human CD3/anti-human CD28 antibodies (STEMCELL Technologies). The presence of cytokines in supernatants was measured using Milliplex kits (Millipore), following the manufacturer’s instructions.

### In vitro HIO-derived enteroid culture (M cell induction)

#### HIO-derived enteroid preparation and in vitro expansion

At 20 weeks post-transplantation, a section of transplanted HIOs was used to isolate the crypts following our protocol for human intestinal tissue^38^. Briefly, the mucosal layer from portions of transplanted HIOs was dissected under a microscope and scraped to remove the villi and debris. The mucosa was then incubated with freshly prepared 2 mM EDTA solution and gently shaken for 30 min. After several washes with ice-cold chelation buffer, the intestinal crypts were collected by gently scraping the mucosa with curved forceps and filtered twice through a 150-μm nylon mesh to remove any debris. Due to limited amounts of tissues, the crypts collected from each group were pooled. The crypts were then washed in ice-cold chelation buffer and 50 μl of crypts, resuspended in Matrigel (Corning), was added per well in a 24-well plate. After polymerization of the Matrigel, 500 μl of human IntestiCult Organoid Growth medium (STEMCELL Technologies) was added to each well. The crypts were cultured and expanded for 10–14 d before being frozen down for later use.

#### M cell induction in vitro

Enteroids were plated on Transwells as described previously^7,39^. Briefly, after being washed from Matrigel, enteroids were fragmented and plated on human collagen IV-precoated 24-well Transwells (0.4-μm pore size) and incubated in IntestiCult Organoid Growth medium (STEMCELL Technology) at 37 °C until monolayer confluence was reached. To induce M cell differentiation, enteroid monolayers were cultured in differentiation medium supplemented with 50 ng ml^−1^ TNF-α and 100 ng ml^−1^ RANK-L for a period of 5 d, as previously described^39^.

### Histology staining

#### IHC and immunofluorescence staining

HIO and mouse SI tissues were fixed overnight in 4% PFA at 4 °C, paraffin embedded and sectioned at 5 μm. For human CD3, CD20, CD4, CD8 and MUM1 staining, the slides were prepared by the Pathology Core at CCHMC using Automation VENTANA BenchMark instruments (Supplementary Table). For HIO staining, sections were prepared as previously described^13^. Briefly, sections were deparaffinized, subjected to antigen retrieval in Dako solution pH 6 (citric acid), permeabilized in 0.5% TritonX in PBS, blocked for 1 h at room temperature in PBS/1% BSA supplemented with serum and then incubated overnight at 4 °C with primary antibody diluted in PBS/1% BSA. The next day, slides were washed and incubated overnight at 4 °C with biotinylated or AlexaFluor-conjugated secondary antibody diluted in PBS/1% BSA. Signals were amplified with RTU Vectastain ABC reagent (Vectorlab) and precipitated using DAB Kit solution (Vectorlab), and finally counterstained with Mayer’s hematoxylin solution (Dako). Images were captured on a Nikon Eclipse Ti and analyzed using Nikon Elements Imaging software (Nikon).

#### Immunofluorescence staining on human fetal intestinal tissue

Immunofluorescence staining was conducted as previously described^40^. Briefly, human fetal intestinal tissue (~0.5-cm fragments) was fixed in 10% neutral buffered formalin for 24 h at room temperature, then paraffin embedded and sectioned (5-µm thickness). Paraffin sections were first deparaffinized in Histo-Clear II (National Diagnostics) and re-hydrated. Antigen retrieval was performed by steaming slides in a sodium citrate buffer for 20 min. Slides underwent a blocking step using 5% normal donkey serum (diluted in PBS + 0.5% Tween20) for 1 h at room temperature. Human CDH1 and CD45 primary antibodies were diluted 1:500 in blocking solution and slides were incubated with antibodies overnight at 4 °C (Supplementary Table). The following day, slides were washed and incubated with secondary antibodies (1:500) diluted in a blocking buffer for 1 h at room temperature, together with DAPI staining (1 μg ml^−1^). Slides were washed and mounted using Prolong Gold (Thermo Fisher). Imaging was done using a Nikon A1 confocal at the University of Michigan Medical School and images were assembled using Photoshop CC. Images were adjusted in Photoshop to optimize for visualization. For all images, any post-image processing (that is, pseudocoloring, brightness, contrast, lookup tables) was performed equally on entire images from a single experiment.

#### Immunofluorescence confocal imaging on HIO-derived enteroids

HIO-derived enteroid monolayers were fixed in aqueous 4% PFA (Electron Microscopy Sciences) for at least 30 min at room temperature, as previously described^7^. Briefly, fixed monolayers were washed with PBS followed by simultaneous permeabilization and blocking in a solution of 15% FBS, 2% BSA and 0.1% saponin (Sigma-Aldrich) in PBS for 30 min at room temperature. Cells were rinsed with PBS and incubated overnight at 4 °C with primary mouse monoclonal antibody to human GP2 diluted 1:100 in PBS containing 15% FBS and 2% BSA. Stained cells were then washed three times for 10 min each with PBS followed by secondary antibody diluted 1:100 in PBS. Probes including phalloidin (AlexaFluor 633) and Hoechst 33342 (Invitrogen) for nuclear/DNA labeling were used at a 1:1,000 dilution in PBS. After incubation, cells were washed three times for 10 min each and mounted in ProLong Gold (Vector Laboratories) overnight at 4 °C.

### RNA extraction and qPCR

#### Tissues

Transplanted HIOs and humanized mouse SI were lysed in RLT buffer from RNAeasy Mini Kit (Qiagen) and RNA was extracted following the manufacturer’s instructions. RNA (500 ng) was used in complementary DNA synthesis using High-Capacity cDNA Reverse Transcription Kit (Applied Biosystems) following the manufacturer’s standard protocol. Gene expression was evaluated using TaqMan gene expression assays (Applied Biosystems) and performed on a StepOne Plus Real-Time PCR System (Applied Biosystems). Primers will be provided upon request.

#### Enteroids

Medium was aspirated from monolayers and both basolateral and apical sides were washed once with PBS. Ambion PureLink RNA Mini Kit lysis buffer was added to each well per the manufacturer instructions. The buffer was used to gently dislodge the monolayer. The three lysis washes were collected in a 15-ml conical for RNA extraction. RNA was quantified with the Qubit RNA HS Assay (Life Technologies). Then, 1.0 μg of RNA was used in cDNA synthesis using Superscript IV Reverse Transcriptase (Life Technologies) following the manufacturer’s standard protocol. Gene expression was evaluated using IDT PrimeTime qPCR Assays (IDT) following both the protocol and suggested cycling conditions for 10-μl reactions. qPCR was performed on the QuantStudio 12K Flex Real-Time PCR System (Applied Biosystems) and analyzed with the QuantStudio 12K Flex software v.1.2.2 (Applied Biosystems). Primers will be provided upon request.

### Human IgA enzyme-linked immunosorbent assay

Mucus samples from HIOs injected with saline or *E. coli* were collected using a 0.5-ml insulin syringe and kept at −80 °C until ready to be analyzed by enzyme-linked immunosorbent assay (ELISA). Following the manufacturer’s instructions, samples were diluted and loaded on a precoated plate from the Human IgA ELISA Kit (Invitrogen). After incubation, results were read at 450 nm on a Synergy H1 microplate reader (BioTek).

### Statistical analysis

All of the data are presented as mean ± s.d. or as mean ± s.e.m. and were analyzed using Prism software (GraphPad). Statistical significance of differences was assessed using multiple Mann–Whitney tests to compare independent samples such as for HIO growth or treatment (saline versus *E. coli*), or using Wilcoxon matched-pairs signed rank tests to compare paired samples such as for immune cells from each humanized mouse isolated from HIOs or mouse SI. The significance cutoff was *P* < 0.05.

### Reporting summary

Further information on research design is available in the Nature Portfolio Reporting Summary linked to this article.

## Online content

Any methods, additional references, Nature Portfolio reporting summaries, source data, extended data, supplementary information, acknowledgements, peer review information; details of author contributions and competing interests; and statements of data and code availability are available at 10.1038/s41587-022-01558-x.

## Supplementary information

Supplementary Table List of antibodies.

Reporting Summary

## Data Availability

Data are available on FlowRepository with ID no. FR-FCM-Z3L6. All the analysis code has been deposited to GitHub: https://github.com/praneet1988/Analyze_CyTOF_Using_Seurat.
