# Supplementary material for: In vivo development of immune tissue in human intestinal organoids transplanted into humanized mice
Source: Nat Biotechnol. 2023 Jan 26;41(6):824–31. doi: 10.1038/s41587-022-01558-x (PMC10264243; doi:10.1038/s41587-022-01558-x)
Supplement: Supplementary file 1 — List of antibodies. [file 41587_2022_1558_MOESM1_ESM.pdf]

nature biotechnology

Article

https://doi.org/10.1038/s41587-022-01558-x

In vivo development of immune tissue in human intestinal organoids transplanted into humanized mice

In the format provided by the authors and unedited

Supplementary Table.

Primary Ab	Company	Dilution	Catalog #
human CD45	Origene	1:800 (IHC) or 1:500 (IF)	SM1744P
human CDH1 (E-Cadherin)	R&D	1:500	AF748
human CD3	Roche	RTU	790-4341
human CD20 (for IHC)	Roche	RTU	760-2531
human CD20 (for IF)	Abcam	1:250	ab64088
human CD4	Roche	RTU	790-4423
human CD8	Roche	RTU	790-4460
human MUM-1	Roche	RTU	760-4529
human GP2	MBL	1:250 (IHC) or 1:200 (IF)	D277-3
human Villin	ThermoFisher	1:100	MA5-12227
human Mucin 2	Abcam	1:200	ab134119
human Lysozyme	Biorad	1:2000	0100-0523
human Chromogranin A	DSHB	1:500	CPTC-CHGA-1
FITC-conjugated anti-human CD45	Biolegend	1:200	368507
PE-Cy5-conjugated anti-mouse CD45	eBioscience	1:200	15-0451-81
BV650-conjugated anti-human CD19	Biolegend	1:200	302237
PE-Cy7-conjugated anti-human CD3	Biolegend	1:200	300316
BV421-conjugated anti-human CD56	Biolegend	1:200	362551
PE-conjugated anti-human CD13	Biolegend	1:200	301703
PE-conjugated anti-human CD33	Biolegend	1:200	366607
APC-Cy7 conjugated anti-human CD4	Biolegend	1:200	344615
PerCP-Cy5.5 conjugated anti-human IL-2	Biolegend	1:150	500321
BV711 conjugated anti-human TNFa	Biolegend	1:150	502939
BV510 conjugated anti-human IFNg	Biolegend	1:150	502543
donkey anti-Rat	Jackson ImmunoResearch	1:500	712-165-153
donkey anti-goat	Jackson ImmunoResearch	1:500	705-605-147
donkey anti-Rat alexa fluor 488	ThermoFisher	1:1000	A-21208
donkey-anti-goat alexa fluor 647	ThermoFisher	1:1000	A-21447
donkey anti-mouse alexa fluor 568	ThermoFisher	1:1000	A10037
goat anti-rabbit alexa fluor 555	ThermoFisher	1:1000	A-10040
UltraView Universal DAB	Roche	RTU	760-500
UltraView Universal AP Red	Roche	RTU	760-501
Y89 Mouse CD45	Fluidigm	1:200	3089005B
Pr141 Human CCR6	Fluidigm	1:200	3141003A
Nd142 Human CD19	Fluidigm	1:200	3142001B
Nd143 Human CD117	Fluidigm	1:200	3143001B
Nd145 Human CD4	Fluidigm	1:200	3145001B
Nd146 Human CD8a	Fluidigm	1:200	3146001B

Sm147 Human CD11c	Fluidigm	1:200	3147008B
Sm149 Human CD56	Fluidigm	1:200	3149021B
Eu151 Human CD103	Fluidigm	1:200	3151011B
Sm152 Human TCRg/d	Fluidigm	1:200	3152008B
Sm154 Human CD45	Fluidigm	1:200	3154001B
Gd155 Human CD45RA	Fluidigm	1:200	3155011B
Gd158 Human CD45RO	Biolegend	1:200	304239
Tb159 Human CCR7	Fluidigm	1:200	3159003A
Gd160 Human CD14	Fluidigm	1:200	3160001B
Dy163 Human CRTH2	Fluidigm	1:200	3163003B
Ho165 Human CD16	Fluidigm	1:200	3165001B
Er167 Human CD27	Fluidigm	1:200	3167002B
Er168 Human CD127	Fluidigm	1:200	3168017B
Tm169 Human CD25	Fluidigm	1:200	3169003B
Er170 Human CD3	Fluidigm	1:200	3170001B
Yb171 Human CD20	Fluidigm	1:200	3171012B
Yb173 Human HLA-DR	Fluidigm	1:200	3173005B
Yb176 Human TCRa/b	Fluidigm	1:200	3176015B
Bi209 Human CD11b	Fluidigm	1:200	3209003B
