# Supplementary material for: In vivo development of immune tissue in human intestinal organoids transplanted into humanized mice
Source: Nat Biotechnol. 2023 Jan 26;41(6):824–31. doi: 10.1038/s41587-022-01558-x (PMC10264243; doi:10.1038/s41587-022-01558-x)
Supplement: Supplementary file 2 — Reporting Summary [file 41587_2022_1558_MOESM2_ESM.pdf]

nature research

Corresponding author(s): Michael A. Helmrath

Last updated by author(s): Sep 24, 2022

Reporting Summary

Nature Research wishes to improve the reproducibility of the work that we publish. This form provides structure for consistency and transparency in reporting. For further information on Nature Research policies, see our Editorial Policies and the Editorial Policy Checklist.

Statistics

For all statistical analyses, confirm that the following items are present in the figure legend, table legend, main text, or Methods section.

n/a	Confirmed	
☐	☒	The exact sample size (*n*) for each experimental group/condition, given as a discrete number and unit of measurement
☐	☒	A statement on whether measurements were taken from distinct samples or whether the same sample was measured repeatedly
☐	☒	The statistical test(s) used AND whether they are one- or two-sided<br>*Only common tests should be described solely by name; describe more complex techniques in the Methods section.*
☒	☐	A description of all covariates tested
☐	☒	A description of any assumptions or corrections, such as tests of normality and adjustment for multiple comparisons
☐	☒	A full description of the statistical parameters including central tendency (e.g. means) or other basic estimates (e.g. regression coefficient) AND variation (e.g. standard deviation) or associated estimates of uncertainty (e.g. confidence intervals)
☐	☒	For null hypothesis testing, the test statistic (e.g. F, t, r) with confidence intervals, effect sizes, degrees of freedom and P value noted<br>*Give P values as exact values whenever suitable.*
☒	☐	For Bayesian analysis, information on the choice of priors and Markov chain Monte Carlo settings
☒	☐	For hierarchical and complex designs, identification of the appropriate level for tests and full reporting of outcomes
☒	☐	Estimates of effect sizes (e.g. Cohen's d, Pearson's r), indicating how they were calculated

Our web collection on statistics for biologists contains articles on many of the points above.

Software and code

Policy information about availability of computer code

Data collection	BD LSR Fortessa flow cytometer with FACSDiva software v9<br>Cytek Aurora flow cytometer<br>Bio-plex Manager 6.1<br>Fluidigm Helios CyTOF/mass cytometer<br>Nikon Eclipse Ti microscope<br>Nikon A1 confocal<br>Applied Biosystems QuantStudio 12K Flew Real-Time PCR System<br>Biotek Synergy H1 microplate reader
Data analysis	GraphPad Prism v9<br>FlowJo v10<br>Bio-plex Manager 6.1<br>Fluidigm debarcoder a feature of CyTOF software v7.0<br>FlowCore R package v3.6.3<br>Seurat R package v3.0.2<br>Nikon Elements Imaging software<br>Photoshop CC<br>Applied Biosystems QuantStudio 12K Flex software v1.2.2<br>https://github.com/praneet1988/Analyze_CyTOF_Using_Seurat<br>Biotek Gen5 software v3.11

For manuscripts utilizing custom algorithms or software that are central to the research but not yet described in published literature, software must be made available to editors and reviewers. We strongly encourage code deposition in a community repository (e.g. GitHub). See the Nature Research guidelines for submitting code & software for further information.

Data

Policy information about availability of data

All manuscripts must include a data availability statement. This statement should provide the following information, where applicable:

- Accession codes, unique identifiers, or web links for publicly available datasets
- A list of figures that have associated raw data
- A description of any restrictions on data availability

Data will be deposited to GEO. Accession codes will be available before publication.

Field-specific reporting

Please select the one below that is the best fit for your research. If you are not sure, read the appropriate sections before making your selection.

☒ Life sciences ☐ Behavioural & social sciences ☐ Ecological, evolutionary & environmental sciences

For a reference copy of the document with all sections, see nature.com/documents/nr-reporting-summary-flat.pdf

Life sciences study design

All studies must disclose on these points even when the disclosure is negative.

Sample size	Experiments with statistical analysis have at least a sample size of n=3
Data exclusions	No data excluded.
Replication	Data are representative of at least 3 independent experiments, except for mass cytometry data (2 independent experiments) and in vitro M cell induction.
Randomization	No randomization required for these data sets.
Blinding	Blinding unnecessary because this study is not part of a clinical trial.

Reporting for specific materials, systems and methods

We require information from authors about some types of materials, experimental systems and methods used in many studies. Here, indicate whether each material, system or method listed is relevant to your study. If you are not sure if a list item applies to your research, read the appropriate section before selecting a response.

Materials & experimental systems

n/a	Involved in the study	
☐	☒	Antibodies
☐	☒	Eukaryotic cell lines
☒	☐	Palaeontology and archaeology
☐	☒	Animals and other organisms
☐	☒	Human research participants
☒	☐	Clinical data
☒	☐	Dual use research of concern

Methods

n/a	Involved in the study	
☒	☐	ChIP-seq
☐	☒	Flow cytometry
☒	☐	MRI-based neuroimaging

Antibodies

Antibodies used

human CD45 Origene 1:800 (IHC) or 1:500 (IF) SM1744P
human CDH1 (E-Cadherin) R&D 1:500 AF748
human CD3 Roche RTU 790-4341
human CD20 Roche RTU 760-2531
human CD4 Roche RTU 790-4423
human CD8 Roche RTU 790-4460
human MUM-1 Roche RTU 760-4529
FITC-conjugated anti-human CD45 Biolegend 1:200 368507
PE-Cy5-conjugated anti-mouse CD45 eBioscience 1:200 15-0451-81
BV650-conjugated anti-human CD19 Biolegend 1:200 302237
PE-Cy7-conjugated anti-human CD3 Biolegend 1:200 300316
BV421-conjugated anti-human CD56 Biolegend 1:200 362551
PE-conjugated anti-human CD13 Biolegend 1:200 301703

April 2020

PE-conjugated anti-human CD33 Biolegend 1:200 366607
APC-Cy7 conjugated anti-human CD4 Biolegend 1:200 344615
PerCP-Cy5.5 conjugated anti-human IL-2 Biolegend 1:150 500321
BV711 conjugated anti-human TNFa Biolegend 1:150 502939
BV510 conjugated anti-human IFNg Biolegend 1:150 502543
human GP2 MBL 1:100 D277-3
phalloidin Alexa Fluor 633 Thermo Scientific 1:1000 21840
donkey anti-Rat Jackson ImmunoResearch 1:500 712-165-153
donkey anti-goat Jackson ImmunoResearch 1:500 705-605-147
goat anti-mouse Alexa Fluor 568 Thermo Scientific 1:100 A-11004
UltraView Universal DAB Roche RTU 760-500
UltraView Universal AP Red Roche RTU 760-501
Y89 Mouse CD45 Fluidigm 1:200 3089005B
Pr141 Human CCR6 Fluidigm 1:200 3141003A
Nd142 Human CD19 Fluidigm 1:200 3142001B
Nd143 Human CD117 Fluidigm 1:200 3143001B
Nd145 Human CD4 Fluidigm 1:200 3145001B
Nd146 Human CD8a Fluidigm 1:200 3146001B
Sm147 Human CD11c Fluidigm 1:200 3147008B
Sm149 Human CD56 Fluidigm 1:200 3149021B
Eu151 Human CD103 Fluidigm 1:200 3151011B
Sm152 Human TCRg/d Fluidigm 1:200 3152008B
Sm154 Human CD45 Fluidigm 1:200 3154001B
Gd155 Human CD45RA Fluidigm 1:200 3155011B
Gd158 Human CD45RO Biolegend 1:200 304239
Tb159 Human CCR7 Fluidigm 1:200 3159003A
Gd160 Human CD14 Fluidigm 1:200 3160001B
Dy163 Human CRTH2 Fluidigm 1:200 3163003B
Ho165 Human CD16 Fluidigm 1:200 3165001B
Er167 Human CD27 Fluidigm 1:200 3167002B
Er168 Human CD127 Fluidigm 1:200 3168017B
Tm169 Human CD25 Fluidigm 1:200 3169003B
Er170 Human CD3 Fluidigm 1:200 3170001B
Yb171 Human CD20 Fluidigm 1:200 3171012B
Yb173 Human HLA-DR Fluidigm 1:200 3173005B
Yb176 Human TCRa/b Fluidigm 1:200 3176015B
Bi209 Human CD11b Fluidigm 1:200 3209003B

Validation

Antibodies used in this study were validated by the manufacturers.
For immunohistochemistry (IHC) staining, antibodies were tested on human tissue as positive control.

Eukaryotic cell lines

Policy information about cell lines

Cell line source(s)	human embryonic stem cell line H1
Authentication	H1 WA-01 from WiCell
Mycoplasma contamination	H1 cell line was negative for mycoplasma. Mycoplasma contamination is routinely tested.
Commonly misidentified lines (See ICLAC register)	No commonly misidentified cell line was used in this study.

Animals and other organisms

Policy information about studies involving animals; ARRIVE guidelines recommended for reporting animal research

Laboratory animals	NSGS mice = Immunodeficient NOD/Scid Il2rg null Tg (hIL3, hGM-CSF and hSCF)
Wild animals	No wild animals used in this study
Field-collected samples	No field-collected samples used in this study
Ethics oversight	All experiments were performed with the approval of the Institutional Animal Care and Use Committee of Cincinnati Children's Hospital Medical Center

Note that full information on the approval of the study protocol must also be provided in the manuscript.

Human research participants

Policy information about studies involving human research participants

Population characteristics	Normal, de-identified developing human fetal tissues were obtained from the University of Washington, Laboratory of Developmental Biology, and all work was approved by the University of Washington and the University of Michigan Institutional Review Board (IRB).<br>Normal adult human jejunum were obtained from patients between the ages of 16 and 25 years old. Informed consent or assent was obtained from all patients and/or parent/legal guardians as appropriate. Human tissue collection was performed with the prior approval of Cincinnati Children's Hospital Medical Center's (CCHMC) IRB.
Recruitment	Fetal tissues from elective abortions<br>Patients undergoing bariatric procedures
Ethics oversight	University of Washington and University of Michigan Institutional Review Board<br>Cincinnati Children's Hospital Medical Center's (CCHMC) Institutional Review Board

Note that full information on the approval of the study protocol must also be provided in the manuscript.

Flow Cytometry

Plots

Confirm that:

☒ The axis labels state the marker and fluorochrome used (e.g. CD4-FITC).

☒ The axis scales are clearly visible. Include numbers along axes only for bottom left plot of group (a 'group' is an analysis of identical markers).

☒ All plots are contour plots with outliers or pseudocolor plots.

☒ A numerical value for number of cells or percentage (with statistics) is provided.

Methodology

Sample preparation	After red blood cell lysis, immune cells isolated from peripheral blood of humanized mice were stained for 30 min on ice with the indicated combination of antibodies.<br>For intracellular cytokine staining, immune cells isolated from HIOs or humanized mouse small intestines were stained for at least 1 hour at room temperature with the indicated combination of antibodies.
Instrument	BD LSR Fortessa<br>Cytek Aurora
Software	FACSDiva v9 for acquisition<br>FlowJo v10 for analysis<br>Cytek SpectroFlo v2.0 software for acquistion
Cell population abundance	No cell sorting was performed in this study
Gating strategy	gating strategy = cells were gated based on FSC/SSC parameters and followed by doublet exclusion.<br>unstained sample was used as negative control and single stained samples were used to set up voltages and compensations parameters.

☐ Tick this box to confirm that a figure exemplifying the gating strategy is provided in the Supplementary Information.
